# Impact of Encapsulation Position in Pickering Emulsions on Color Stability and Intensity Turmeric Oleoresin

**DOI:** 10.3390/foods14030385

**Published:** 2025-01-24

**Authors:** Ahreum Han, Youjin Baek, Hyeon Gyu Lee

**Affiliations:** Department of Food and Nutrition, Hanyang University, 222, Wangsimni-ro, Seongdong-gu, Seoul 04763, Republic of Korea; han7230@hanyang.ac.kr (A.H.); jyyj161126@hanyang.ac.kr (Y.B.)

**Keywords:** Pickering emulsion, encapsulation position, color intensity

## Abstract

The emulsification of natural pigment is a widely utilized strategy to enhance its stability in the food industry. However, high turbidity in emulsions often causes color fading, limiting their application. Here, we developed a comprehensive Pickering emulsion (PE) system to improve the color intensity and stability of turmeric oleoresin (Tur) under various food processing conditions. Specifically, the effects of two encapsulation positions within the PE were compared: the inner oil phase (Tur-IPE) and the outer solid particle layer (Tur-OPE). Lysozyme and carboxymethyl cellulose nanoparticles (NPs) were used as natural solid particle surfactants, with their successful formation confirmed through physical property analysis and FTIR spectroscopy. The optimal oil fraction (φ) for suitable physical properties of PE was determined to be 0.2. Interestingly, Tur-OPE significantly exceeded Tur-conventional emulsions (Tur-CE) and Tur-IPE in terms of color vividness, exhibiting higher redness and lower lightness (*p* < 0.05). During thermal processing at 70 and 90 °C, all emulsions demonstrated significantly enhanced heat resistance, retaining 1.3 to 1.6 times more Tur, respectively, compared to unencapsulated Tur (free Tur) (*p* < 0.05). Furthermore, Tur’s pH instability was significantly overcome by encapsulation in all emulsion systems (*p* < 0.05). During 4 weeks of storage period, Tur-OPE demonstrated the highest retention rates, with the half-life of Tur increasing in the following order: free Tur < Tur-CE < Tur-IPE < Tur-OPE. Thus, we highlighted the important role of encapsulation position in PEs in improving and maintaining the color stability and vividness of natural pigments under various food processing conditions.

## 1. Introduction

Color plays an important role in food selection since it affects customer acceptance as well as sensory perception [1]. Recently, the food field has tried to replace synthetic colorants with natural alternatives in response to the increasing demand for clean labeling [2]. Natural pigments have been known to not only provide various health benefits such as antioxidant, anti-inflammatory, and anti-cancer effects, but they are also considered safer options [3,4,5]. Turmeric oleoresin (Tur), which is derived from *Curcuma longa* L. and contains diverse bioactive characteristics, such as antioxidant and anti-inflammatory effects, is known for its yellow-orange color [6,7]. Moreover, curcumin, the main colorant in Tur, has strong antioxidant activity because of its phenolic hydroxyl group [8].

However, Tur’s application in food has been thus far limited because of its low aqueous solubility and chemical instability [9]. Nanoemulsion was consistently used in recent studies as one of the encapsulation strategies in order to enhance Tur’s solubility and stability; however, nanoemulsion often masks the color of natural pigments because of increased turbidity, leading to color fading [10,11]. For example, black chokeberry pomace extract demonstrated brighter, less red, and more yellow color attributes after encapsulation within emulsions, while its original color as a unencapsulated form was pink and purple [12]. Additionally, increasing natural colorants’ concentration for enhancement in their color attributes can negatively influence the physical and sensory qualities of food. In order to address these problems, recent studies have developed new strategy: a combination of co-pigmentation and emulsion encapsulation to enhance the vividness and stability of natural pigments [13]. However, as co-pigmentation is mainly applicable to anthocyanins, this strategy is not suitable for Tur [14].

Pickering emulsions (PE), replacing synthetic surfactants with natural complexes using food biopolymers like proteins and polysaccharides, have various benefits over conventional emulsions (CE), including reduced toxicity and increased safety [15,16]. Interestingly, PE allows for dual encapsulation, wherein the core material could be incorporated into either the inner oil phase or the outer solid particle layer [17]. This present study proposed that this dual-encapsulation approach, which involves encapsulating Tur within either the inner oil phase (IPE) or the outer solid particle layer (OPE), could potentially help control the color of Tur. Nevertheless, most studies on Tur-encapsulated PE have focused on improving the digestive stability and bioaccessibility of Tur [18,19], resulting in little understanding of how the encapsulation location influences Tur’s color. Furthermore, colorimetric stability evaluation under heat, pH, and storage conditions is crucial for food applications; however, there is limited information on these aspects.

To address these research limitations, we aimed to determine how Tur’s color intensity and stability are affected by its encapsulation position. To the best of our understanding, this is the first comprehensive investigation of how different encapsulation positions impact Tur’s color intensity and stability under various food processing conditions, including thermal, pH, and storage. Our overall aim was to develop a novel strategy to regulate Tur’s color attributes through encapsulation within the OPE or IPE while improving its stability during food processing procedures. In order to do this, we (1) formulated carboxymethyl cellulose (CMC) and lysozyme (Ly) nanoparticles (NPs) (Ly/CMC NPs); (2) evaluated the physical properties and molecular structure of Ly/CMC NPs through Fourier transform infrared (FTIR) spectroscopy analysis; (3) formulated Tur-loaded OPE (Tur-OPE) and Tur-loaded IPE (Tur-IPE) stabilized with Ly/CMC NPs; and (4) analyzed their morphological characteristics, colorimetric properties, thermal stability, pH stability, and storage stability.

## 2. Materials and Methods

### 2.1. Materials

Carboxymethyl cellulose (CMC), lysozyme (Ly), N-(3-Dimethylaminopropyl)-N’-ethylcarbodiimide hydrochloride (EDC), Tween 20^®^, Nile blue A, and Nile red were purchased from Sigma-Aldrich Chemical Co. (St Louis, MO, USA). TUR was obtained from ES food (Gunpo, Republic of Korea). Medium-chain triglyceride (MCT) oil was purchased from a local market (Seoul, Republic of Korea).

### 2.2. Preparation of Ly/CMC NPs

Ly/CMC NPs were prepared by electrostatic interactions according to a previously described method [20]. The NP preparation conditions were optimized through our preliminary experiments. Briefly, 2 mg/mL of Ly and 2 mg/mL of CMC as an optimized concentration were dissolved in distilled water separately with stirring at 25 °C for 4 h. Then, 5 mL of Ly solution was added dropwise to 15 mL of CMC solution with stirring at 1200 rpm. To prepare Tur-loaded Ly/CMC NP (Tur-Ly/CMC NP), TUR ethanol solution was added to the CMC solution. Ly was then added while stirring. The Ly/CMC solution was adjusted to pH 9 with stirring (1 h), followed by heating for 15 min at 80 °C. To enhance peptide bond cross-linking, 10 mg/mL of EDC solution (500 μL) was added.

### 2.3. Physical Properties of Ly/CMC NPs

#### 2.3.1. Particle Size, Polydispersity Index, and Zeta Potential

The particle size, polydispersity index (PDI), and zeta potential (ZP) of all prepared samples were assessed with a Malvern Zetasizer Nano ZS (Malvern Instruments Ltd., Worcestershire, UK) using the dynamic light scattering method at a temperature of 25 ± 1 °C [21].

#### 2.3.2. Entrapment Efficiency

The entrapment efficiency (EE) of TUR-Ly/CMC NP was analyzed as previously mentioned [22]. Briefly, TUR-Ly/CMC NPs were centrifuged for 10 min (10,000× *g*) to remove curcumin precipitates, which were unencapsulated. The supernatant was diluted with ethanol, and the Tur encapsulated within NPs were determined using a Synergy HT Multi-microplate reader at absorbance 428 nm (BioTek Instruments, Winooski, VT, USA). The EE (%) was calculated using Equation (1) as follows:

(1) $$EE\left(\%\right)=\frac{TUR\;encapsulated\;in\;NPs}{Total\;amount\;of\;Tur}\times 100$$

### 2.4. ATR-FTIR of Ly/CMC NPs

Freeze-dried Ly, CMC, Tur, Ly/CMC, and TUR-Ly/CMC were analyzed by a Vacuum FTIR Microscope spectrometer (Vertex-80V/Hyperion2000, Bruker, Bremen, Germany) with minor modifications, according to a previous study [23]. The spectra represented were obtained with a resolution of 8 cm^−1^, with averages of 32 scans, and ranging from 600 to 4000 cm^−1^.

### 2.5. Preparation of Emulsion

To prepare the PE, MCT oil was added dropwise with continuous agitation into the freshly prepared Ly/CMC NP dispersion according to Section 2.2 with 2:8, 4:6, and 6:4 ratios (*w*/*w*) [16]. Using a high-speed homogenizer (WiseMix™ HG15A, Daihan Scientific, Seoul, Republic of Korea), the mixture was homogenized at 10,000 rpm (5 min). The mixture was then ultrasonicated for 30 s at 20% amplitude in an ice bath.

TUR-loaded emulsions were prepared by adding TUR (30 μg/g) to either Ly/CMC NP or MCT, followed by emulsification as aforementioned. The PEs containing TUR in the solid particle layer were called outer-layer PE (OPE), whereas those containing TUR in the oil phase were named inner-phase PE (IPE). A conventional emulsion (CE) was prepared using the same procedure with an equal amount of Tween 20 instead of Ly/CMC NP.

### 2.6. Morphological Characterization of PE Stabilized by Ly/CMC NPs

The microstructure of the emulsion droplets and the interfacial adsorption behavior of Ly/CMC NPs in PE were further analyzed by a confocal laser microscope (TCS SP5, Leica, Wetzlar, Germany) based on a previously reported method [24]. MCT oil and Ly/CMC NPs were stained with Nile red and Nile blue A, respectively. The laser excitation wavelengths of Nile red and Nile blue A were 488 nm and 633 nm, respectively. LAS X software (LAS X 1.8.013370, Leica, Wetzlar, Germany) was used to analyze the confocal images. At least 50 droplets were randomly selected from each image and analyzed to determine the average droplet size.

### 2.7. Color Analysis of OPE and IPE

In order to measure the color attributes of samples, a Minolta Chroma Meter (CR-400; Minolta Co., Ltd., Osaka, Japan) was employed. L*, a*, and b* values indicate lightness, redness, and yellowness, respectively [25]. Using a white plate with a* = 0.07, b* = 2.33, and L* = 96.94, the colorimeter underwent calibration before the experiment. The total difference in color attributes were demonstrated as ΔE, which was calculated following Equation (2):

(2) $$\Delta E=\sqrt{{\left({\Delta L}^{*}\right)}^{2}+{\left(\Delta a^{*}\right)}^{2}+{{(\Delta b}^{*})}^{2}}$$

Photographs of all the samples were taken using a digital camera (Galaxy 23; Samsung, Suwon, Republic of Korea).

### 2.8. Stability of the OPE and IPE

#### 2.8.1. Thermal Stability

Unencapsulated Tur (free Tur), Tur-OPE, Tur-IPE, and Tur-CE’s thermal stability was investigated by measuring color attributes and retention rate (%) [25]. Both the free Tur and Tur-loaded emulsions underwent thermal treatment at 70, 80, and 90 °C (1 h). Color attributes were measured as described in Section 2.7. For retention rate measurement, the samples were gathered at 0, 15, 30, 45, and 60 min during thermal treatment, and then, the initial and residual content of Tur within samples were determined using a Synergy HT Multi-microplate reader at absorbance 428 nm (BioTek Instruments, Winooski, VT, USA). The Tur’s retention rate was determined using Equation (3):

(3) $$Retention\;rate\left(\%\right)=\frac{residual\;content\;of\;TUR}{initial\;content\;of\;TUR}\times 100$$

#### 2.8.2. pH Stability

Free Tur and Tur-loaded emulsion samples were prepared to analyze the pH effects on Tur stability. Using either 0.1 M HCl or 1 M NaOH, the pH was adjusted to various levels within the range of 3–9 at 25 °C. The color was then evaluated as previously described.

#### 2.8.3. Storage Stability

To study long-term stability, the samples were stored for 4 weeks in the dark at 25 °C. The effects of storage on the color and retention rate were analyzed as previously described. Using a first-order kinetic model, Tur degradation was calculated [26]. The half-life (*t*_1/2_) of TUR and the degradation rate constants (*k*) after storage were calculated via Equations (4) and (5) as follows:

(4) $$-ln\frac{C_{t}}{C_{0}}=kt$$

(5) $$t_{1/2}=\frac{ln2}{k}$$

*C_t_* and *C*_0_ are the TUR contents at times *t* and *t*_0_, respectively. *t* is the storage time.

### 2.9. Statistical Analysis

All experiments were demonstrated as the calculated mean ± standard deviation (SD), with each experiment carried out in triplicate. A one-way analysis of variance was employed to assess the data, followed by Turkey’s test (*p* < 0.05). Statistical analyses were performed using SPSS 27.0 (SPSS Inc., Chicago, IL, USA).

## 3. Results and Discussion

### 3.1. Physical Properties of Ly/CMC NPs

The particle size, PDI, ZP, and EE of Ly/CMC NPs and Tur-Ly/CMC NPs are listed in Table 1. Upon encapsulation of Tur, the particle size of the Ly/CMC NPs increased significantly, whereas the PDI decreased notably to below 0.4 (*p* < 0.05). Despite these changes, the ZP values of both Ly/CMC NPs and Tur-Ly/CMC NPs remained greater than −50 mV, showing no significant difference. The EE of Tur-Ly/CMC NPs was approximately 48.7%.

Ly and CMC can form complexes via electrostatic interactions because of their positive and negative charge, respectively [19]. In our preliminary study, we found that the Ly/CMC NPs’ particle size reduced as the CMC concentration rose. Stronger interactions between the Ly molecules and CMC chains at higher concentration of CMC might account for this phenomenon [20,27]. Interestingly, due to Tur’s occupation of space in the Ly/CMC NPs, the particle size of NPs increased by about 3.7 times [28]. Furthermore, according to Baek et al. (2023), a PDI value of less than 0.3 shows a uniform particle distribution, while a value greater than 0.3 indicates a non-uniform distribution [21]. In this study, both Ly/CMC NPs and Tur-Ly/CMC NPs had PDI values higher than 0.3 because of the variable length of the CMC chain [29]. Nevertheless, the PDI of Ly/CMC NPs decreased after Tur encapsulation, which may be due to the presence of curcumin in Tur [30]. Curcumin could have hydrophobic and electrostatic interactions with Ly/CMC NPs, leading to more uniform particle size distribution [31]. This result was consistent with a previous study, which confirmed a decreased PDI value of Ly/CMC NPs after curcumin encapsulation [29]. ZP values, a surface charge indicator of NPs, demonstrate strong electrostatic repulsion between particles when their absolute values exceed 30 mV [27]. In this study, ZP values of both Ly/CMC NPs and Tur-Ly/CMC NPs were less than −30 mV, indicating high stability.

Although curcumin in Tur could have a strong interaction with Ly/CMC NPs, the EE of Tur-Ly/CMC NPs was less than 50%, while a previous study showed higher EE than that in this present study [29]. The differences in the preparation methods may explain this reduced efficiency. Tur-Ly/CMC NPs, prepared in this present study, were heated at 80 °C for 15 min, whereas a previous study did not involve any heating process. During the heating process, the degradation of the curcumin in Tur might have occurred, leading to a lower EE [30]. Despite this, we confirmed that Tur was successfully encapsulated within Ly/CMC NPs, maintaining a small particle size and high electrostatic stability for further application in PE.

### 3.2. FTIR Analysis of Ly/CMC NPs

**Figure 1:**
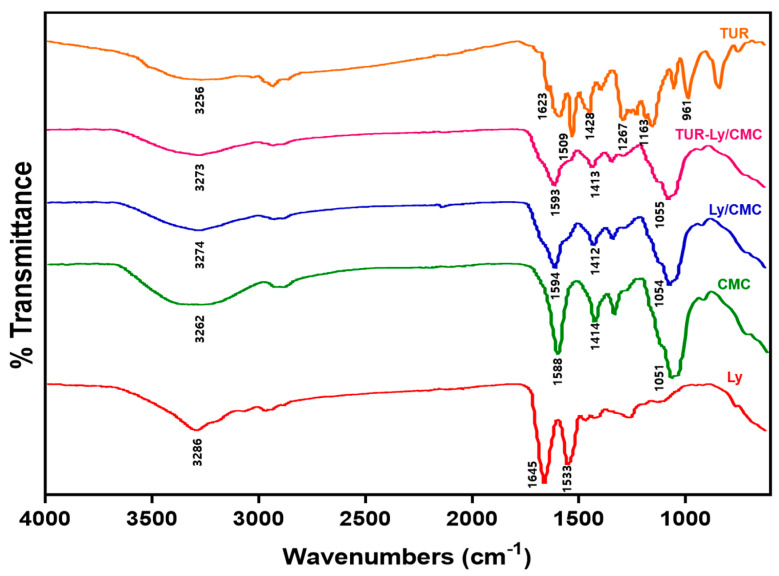
FTIR spectra of Ly, CMC, Ly/CMC, TUR-Ly/CMC, and TUR. Ly, lysozyme; CMC, sodium carboxymethyl cellulose; Tur, unencapsulated turmeric oleoresin; Ly/CMC NPs, lysozyme-sodium carboxymethyl cellulose nanoparticles; Tur-Ly/CMC NPs, turmeric oleoresin lysozyme-sodium carboxymethyl cellulose nanoparticles.

For the purpose of analyzing the Ly, CMC, and free Tur interactions and structural changes at the molecular levels, FTIR spectroscopy is an effective method. As shown in Figure 1, the bond interactions in free Tur, Ly, CMC, and Ly/CMC NPs were analyzed through FTIR spectroscopy. A peak between 3200 and 3300 cm^−1^, which indicates the presence of hydrogen bonding, was demonstrated in the spectra of free Tur, Ly, and CMC [23].

In the case of Ly, the peak at 1645 cm^−1^ and peak at 1533 cm^−1^ show the stretching of the C=O group (amid I band) and the combination of N-H bending and C-N stretching (amid II band), respectively [32]. In the case of CMC, the C=O stretching vibration and symmetrical stretching vibrations of the carboxyl groups were illustrated in the peaks at 1588 cm^−1^ and 1414 cm^−1^, respectively [33]. Moreover, the peak at 1051 cm^−1^ indicates the C–O–C stretching vibrations [34].

Interestingly, the spectra of Ly/CMC NPs exhibited the main peaks from both Ly and CMC at 1000–1650 cm^−1^, including the C=O (1645 and 1588 cm^−1^), N-H (1533 cm^−1^), carboxyl (1414 cm^−1^), and C-O-C (1051 cm^−1^) groups, with reduced intensities. This phenomenon suggests that the amine group in Ly and carbonyl group in CMC interacted through electrostatic and hydrogen bonds during Ly/CMC NPs formation [35].

In the spectrum of free Tur, C=O stretching was demonstrated in the peak at 1623 cm^−1^, and the C-C vibration and olefinic bending vibration of C-H bonded to the benzene ring were exhibited in the peaks at 1509 cm^−1^ and 1428 cm^−1^, respectively [36,37]. Peaks at 1163 cm^−1^ and 1267 cm^−1^ demonstrated evidence of C-O-C stretching vibrations and C=O stretching vibrations associated with the aromatic ring, respectively [38]. Furthermore, the peak at 961 cm^−1^ was associated with the enol moiety’s hydroxl group [39]. The FTIR results of free Tur in this study were consistent with a previous study that analyzed the FTIR spectrum of curcumin [38]. Moreover, curcumin exists in a keto-enol tautomer form, as evidenced from the absence of a peak at 1800–1650 cm^−1^ (carbonyl region) in the FTIR spectrum of free Tur [40].

In the case of Tur-Ly/CMC NPs, the main peaks in Ly/CMC NPs were consistently observed, while Tur’s characteristic peaks were not. This indicates that incorporation of Tur within Ly/CMC NPs successfully occurred. A previous study reported a similar finding, in which the disappearance of main peaks in blackberry juice after encapsulation within a protein and polysaccharide matrix resulted from successful encapsulation [41]. Thus, our study demonstrates that Ly interacts with CMC to form self-assembled NPs that effectively protect Tur through encapsulation.

### 3.3. Morphological Characteristics of PE Stabilized by Ly/CMC NPs

**Figure 2:**
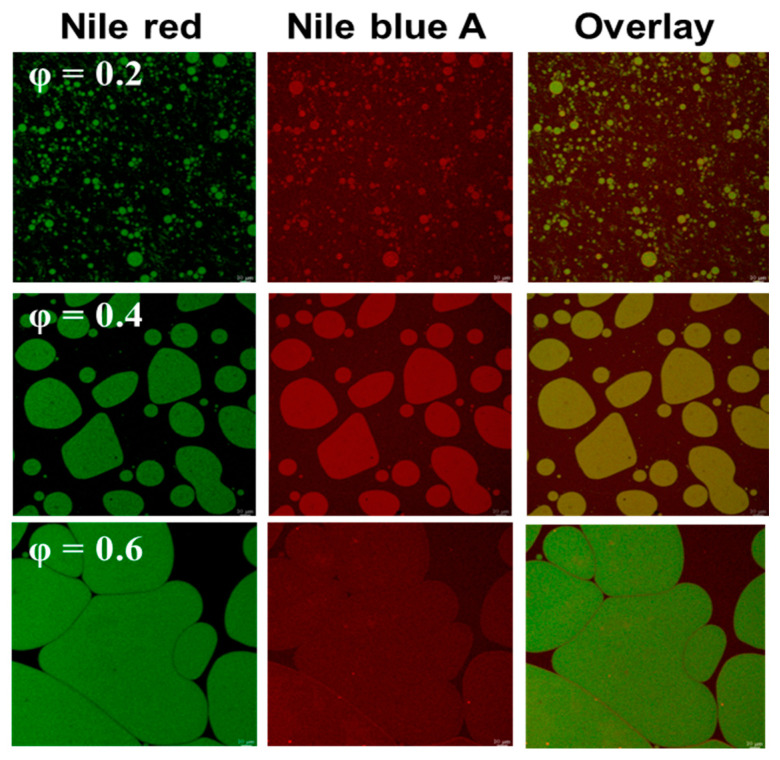
CLSM images of PE stabilized by Ly/CMC NPs with various oil fraction (φ). CLSM images of PE, scale bar 10 μm. PE, Pickering emulsion.

The microstructure of PE stabilized by Ly/CMC NPs at different oil fractions were analyzed by the CLSM images (Figure 2). From the CLSM images, we revealed that oil droplets were surrounded by Ly/CMC NPs, forming a stable oil-in-water structure. In general, solid particles, such as proteins and polysaccharide complexes, could be employed to stabilize PE by being absorbed in the oil–water interface [42]. For instance, Xu and his coworkers reported that Ly and xanthan gum complexes effectively prevented oil droplet coalescence by being strongly absorbed in the oil–water interface [16]. Similarly, the Ly/CMC NPs prepared in this study, could stabilize PE through dense absorption at the oil–water interface.

Importantly, particle size was increased along with an increment in size non-uniformity as φ increased, suggesting that φ greatly affects PE’s particle size and morphology. Small and spherical oil droplets were observed in the PE at φ = 0.2, while larger and irregularly shaped droplets formed at φ greater than 0.4. This indicates that the PE became unstable owing to insufficient Ly/CMC NPs at φ levels higher than 0.4, as they were unable to properly absorb at the oil–water interface and retain a spherical oil droplet shape [43]. Hence, it would be essential to precisely determine the optimal φ—0.2 in this study—for Ly/CMC NPs to effectively stabilize PE. Additionally, the PE particle size at φ = 0.2 and 0.4 was found to be 4.04 ± 2.05 and 14.88 ± 14.70 μm, respectively. However, due to reduced stability, such as coalescence and oiling-off, the particle size at φ = 0.6 could not be determined. These results demonstrated that small and uniform particle size with a spherical morphology of PE stabilized by Ly/CMC NPs was obtained at φ = 0.2. Thus, PE at φ = 0.2 was chosen for further analysis.

### 3.4. Color Properties of OPE and IPE

**Figure 3:**
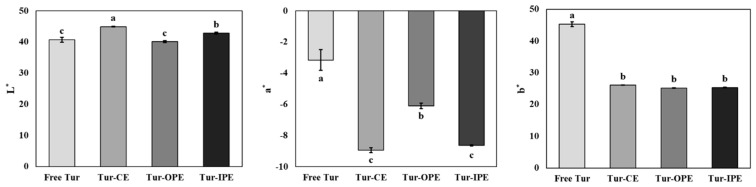
Color attributes (L*, a*, and b*) of free Tur and Tur-loaded emulsions. Free Tur, unencapsulated turmeric oleoresin; Tur-CE, turmeric oleoresin-loaded conventional emulsion; Tur-OPE, Pickering emulsion encapsulating turmeric oleoresin in its outer layer; Tur-IPE, Pickering emulsion encapsulating turmeric oleoresin in its inner phase. ^(a–c)^ Different letters indicate significant differences with *p* < 0.05.

As illustrated in Figure 3, the color values (L*, a*, and b*) of Tur-loaded emulsions (Tur-OPE, Tur-IPE, and Tur-CE) were significantly influenced by encapsulation position (*p* < 0.05). When Tur was encapsulated within the all emulsion samples, L* increased, whereas both a* and b* decreased significantly compared with those of free Tur (*p* < 0.05). Among different emulsion formulations, the L* values followed the order Tur-CE > Tur-IPE > Tur-OPE. Additionally, when Tur was encapsulated within the inner oil phase of the emulsion, Tur-CE and Tur-IPE in this study, a* values significantly decreased compared to those of Tur-OPE (*p* < 0.05), whereas b* values did not show any significant differences between the encapsulation locations of Tur.

In general, the scattering and absorption characteristics could influence the color attributes of the emulsion. Specifically, the turbidity, opacity, and brightness of emulsion are affected by its scattering properties, while its chromaticity is determined by its absorption characteristics [44]. Our present study proved the impact of emulsion encapsulation on color attributes, as evidenced by the increased lightness and decreased redness of Tur after encapsulation within CE, likely due to its contribution to enhanced turbidity [11].

Interestingly, a significant difference in lightness (L* values) between Tur-CE and Tur-IPE, which encapsulated Tur in the same inner phase position, was observed (*p* < 0.05), whereas the a* and b* values between them were not notably different. This phenomenon could be explained by the type of surfactant used to prepare the emulsion. In the case of Tur-CE, tween 20 formulated a clear boundary between the oil and water phases, allowing more light to scatter as it passed through, thereby, leading to a lighter emulsion [45]. In contrast, Ly/CMC NPs as a Tur-IPE stabilizer generated a rigid layer between the oil and water phases, which reduced light scattering, thereby resulting in a darker emulsion (lower L* values) [46]. Tur-OPE, with Tur encapsulated within Ly/CMC NPs, showed significantly higher redness (high a* value) and lower lightless (low L* value) than that of Tur-CE and Tur-IPE (*p* < 0.05). When Tur is positioned in the outer layer of PE, it interacts more with light, leading to enhanced color vividness and redness. Additionally, a deeper and more reddish appearance of Tur-OPE could also be explained by the pH adjustment to a basic level during the preparation process of Tur-Ly/CMC NPs, which would lead Tur to change color toward orange [47].

Based on these results, we verified differentiating encapsulation position as a promising strategy for addressing the issue of emulsions with the reduced color intensity of natural pigments to broaden their food applications. Thus, stabilizing PE with natural colorants like Tur-loaded Ly/CMC NPs could enhance the color intensity, thereby improving the color properties.

### 3.5. Stability of OPE and IPE

#### 3.5.1. Thermal Stability

As food quality could be negatively influenced by the natural pigment degradation, it is crucial to maintain the color attributes during food processing. As illustrated in Table 2, we investigated how the color attributes of free Tur, Tur-CE, Tur-OPE, and Tur-IPE changed during thermal treatment at 70 and 90 °C. In the case of thermal treatment at 70 °C, no significant differences were observed in ΔL* between free Tur and all emulsion samples. On the other hand, free Tur showed significantly higher values in the Δa* and Δb* than those for all emulsion samples (*p* < 0.05). Additionally, free Tur exhibited a 2-fold higher ΔE value, as a total color change indicator, than that for emulsion samples, with significant differences (*p* < 0.05). Given that ΔE is less than 2, which indicates that the color change can only be detected by close observation, our result suggests that the overall color change of free Tur is easy to identify, while that of all emulsion samples is not [48]. Additionally, there were more noticeable changes in all color attributes when the treatment temperature increased to 90 °C.

**Figure 4:**
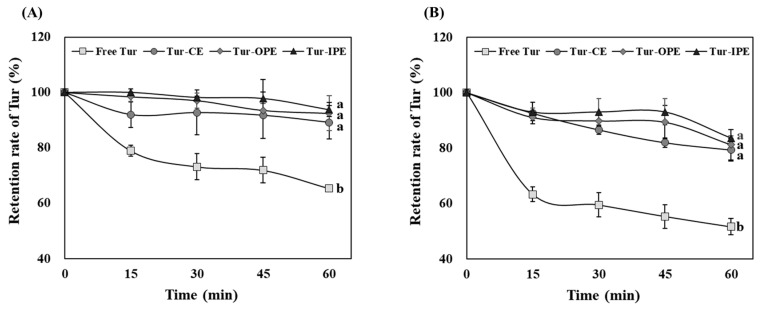
Effect of heating temperature on retention rate (%) of free Tur and Tur-loaded emulsions at (**A**) 70 and (**B**) 90 °C. Free Tur, unencapsulated turmeric oleoresin; Tur-CE, turmeric oleoresin-loaded conventional emulsion; Tur-OPE, Pickering emulsion encapsulating turmeric oleoresin in its outer layer; Tur-IPE, Pickering emulsion encapsulating turmeric oleoresin in its inner phase. ^(a–b)^ Different letters indicate significant differences at *p* < 0.05.

Along with the color attributes change results, the retention rates of free Tur and emulsion samples during heat treatment was analyzed (Figure 4). After thermal treatment either at 70 or 90 °C, the retention rate of free Tur significantly decreased compared to that of emulsion samples (*p* < 0.05). On the other hand, the emulsion samples did not demonstrate significant differences in their retention rates regardless of heating temperature. This implies that Tur, regardless of where it is encapsulated within the PE, became more stable through encapsulation within PE.

Because of the diketone bond structure that is prone to breakdown at high temperatures, curcumin, the primary bioactive compound in Tur, demonstrates low thermal stability [30]. Our present study showed a consistent result from a previous study, which found that heat treatment resulted in a decrease in the retention rate of free curcumin [31]. Tur, on the other hand, exhibited improved heat stability when encapsulated within emulsion systems, as evidenced by the low color changes and comparatively high retention rates. The enhanced thermal stability of Tur-loaded PE could be attributed to the protective barrier formed by Ly/CMC NPs. These NPs likely prevent the breakdown of curcumin by providing steric and electrostatic repulsion that inhibits droplet aggregation and leakage during heating [49]. In other words, the heat resistance characteristics of Ly/CMC NPs are responsible for the increased thermal stability demonstrated in the Tur-IPE and Tur-OPE, comparable to that in the CE [50]. Because Ly and CMC complexes might form gel-like structures at elevated temperatures, these NPs could prevent PE from being broken down [51]. Thus, the ability of Ly/CMC NPs to prevent Tur leakage from the PE accounts for the remarkable stability of both Tur-OPE and Tur-IPE, irrespective of Tur encapsulation location.

In turn, the PE stabilized by Ly/CMC NPs in this study demonstrated high thermal stability similar to that of CE and retained Tur’s color characteristics regardless of encapsulation position alternations. The encapsulation position-differentiating approach established in this present study enabled the retention of Tur’s various color intensities, i.e., brighter and less vivid for Tur-IPE and darker and more vivid for Tur-OPE, even during the heat process, leading to enhanced applicability in the food industry.

#### 3.5.2. pH Stability

To assess the effects of Tur encapsulation position on its applicability in the food industry, pH stability was evaluated at pH 3, 5, 7, and 9 (Table 3). At pH 3, 7, and 9, free Tur exhibited significantly higher ΔL* and Δb* values and significantly lower Δa* values compared to those of the emulsion systems (*p* < 0.05). Additionally, the ΔE values for free Tur were 7.3–13.9 times higher at pH 3, 3.2–4.7 times higher at pH 7, and 8.8–10.3 times higher at pH 9 than those of all emulsion systems. These results indicate that free Tur became darker and more yellow, while its redness decreased at pH 3, 7, and 9. In contrast, all the emulsion systems showed minimal color changes, with no significant differences in color attributes among the samples. In summary, Tur-OPE maintained its dark and reddish color attributes across all pH levels, whereas Tur-CE and Tur-OPE preserved their brighter and more yellow color attributes.

Generally, curcumin is found in a keto-enol tautomeric form that, depending on the pH, can alternate between ketone and alcohol structure [30]. Due to its limited solubility, curcumin can precipitate at low pH levels and tends to deteriorate more quickly at neutral or higher pH values, which causes color fading [52]. In contrast, the emulsion systems demonstrated less color change at pH 3 and 9 compared to that of free Tur. This suggests that encapsulating Tur within an emulsion creates a physical barrier that enhances pH stability. As previously mentioned, the enhanced stability of PE, which is comparable to that of CE, indicates that Ly/CMC NPs, despite being natural solid particle emulsifiers, offer a similar improvement in pH stability as Tween 20, a synthetic emulsifier.

There are two main reasons for the high pH stability of Ly/MC NPs in the pH range of 3–9. First, Ly does not change significantly with pH unless in extremely alkaline environments. At pH values above 11, structural loosening would occur when Ly’s secondary structure changes from an α-helix to a β-sheet [53]. This structural alteration may influence the integrity of Ly/CMC NPs negatively, decreasing PE’s emulsion stability and making it more difficult to control the release of Tur from the NPs. Second, the positive and negative charges on Ly and CMC, respectively, cause them to form NPs through electrostatic interactions. The charge of Ly becomes negative at high pH values (pH > 10), while the charge of CMC approaches zero at low pH values (pH < 2) [54]. In conclusion, Ly and CMC maintained opposing charges at pH 3–9, which increased the NPs’ stability. Nevertheless, the charges of CMC and Ly changed to either a positive or negative value outside of this pH range, which could cause the NPs to dissociate. According to a previous study, PE prepared with NPs from Ly and xanthan gum demonstrated high stability throughout a comparable pH range, except extremely alkaline conditions [54].

Overall, in this study, Tur-OPE retained its intense reddish color across various pH levels, whereas Tur-IPE maintained its light-yellow color. This indicates that the encapsulation of Tur in different positions within the PE can achieve the desired color stability, even under different pH conditions in food applications.

#### 3.5.3. Storage Stability

As shown in Table 4, we evaluated the color attribute changes of free Tur, Tur-CE, Tur-OPE, and Tur-IPE during 4 weeks of storage. There were no notable differences in the ΔL* values among all emulsion samples and free Tur. The Δa* and Δb* values for free Tur, on the other hand, were much greater than those of all emulsion samples, suggesting that encapsulating Tur within the emulsion improved the preservation of its color intensity. Furthermore, free Tur had the largest ΔE value, whereas all the emulsion samples had ΔE values less than 2.

Additionally, the retention rate of free Tur and all emulsion samples over the storage period were analyzed in Table 5, which can be employed to further explain the change in color characteristics. Over 4 weeks, the retention rate of free Tur (residual content of Tur versus initial content of Tur) decreased dramatically from 100 to 34% (*p* < 0.05). In contrast, the retention rate of all emulsion samples decreased more slowly, from 100 to about 80%. Among the various emulsion samples, Tur-OPE was the sole emulsion system that did not demonstrate any significant differences during storage, suggesting its high stability. These findings indicated that encapsulation of Tur within CE, OPE, or IPE improved storage stability and decreased leakage, resulting in negligible differences in color attributes. As aforementioned, the color change could only be distinguished by careful observation when the ΔE value is less than 2 [48]. In this context, all emulsion systems had little color changes even after storage (ΔE < 2), due to their great stability, whereas the color change of free Tur could be observed with the naked eye during the storage period (ΔE > 2). In this present study, PE stabilized by Ly/CMC NPs demonstrated color changes and retention properties similar to Tur-CE, indicating the efficacy of Ly/CMC NPs in improving emulsion stability. These results were comparable to the findings regarding thermal and pH stability. According to a previous study, NPs prepared with Ly and polysaccharides exhibited good stability, which might help the emulsion become even more stable [55]. Thus, Tur-OPE retained its reddish color attributes better than Tur-IPE, which had a pale yellow color, even after 4 weeks of storage.

To ensure that the encapsulation system is suitable for utilization in the food industry, it is important to investigate its release characteristics, including its kinetic model and half-life [56]. According to Lee et al. (2024), kinetic models offer significant insights into the patterns of core material release from the encapsulation system [25]. A first-order kinetic model in this investigation with high R^2^ values could explain the release patterns of Tur. This, in turn, suggests that, regardless of Tur’s encapsulation location within the emulsion, the release behavior of Tur from these emulsion samples followed the diffusion-based kinetics observed in a matrix-type encapsulation system [26]. Similar kinetics were observed for curcumin in a previous study when released from hydrogel-incorporated emulsions [57].

Furthermore, the half-life is a critical property for analyzing how well an encapsulation system maintains the core material from external factors [58]. Generally, half-life is the amount of time needed for half of the core material to be released [58]. In the present study, the half-life of free Tur was 18.34 days; however, this was extended by 5.48, 9.00, and 7.41 times through encapsulation within CE, OPE, and IPE, aligning with the trends observed in previous study [19]. When compared to Tur-CE and Tur-IPE, the half-life of Tur-OPE was 65 and 30 days longer, respectively. This result suggests that Tur could remain longer in PEs than in CE when it is encapsulated. Additionally, it was shown that locating Tur in the outer layer of the PE was a better strategy to extend retention than positioning it in the inner oil phase. The reason for this result is that Ly/CMC NPs’ compact structure prevented Tur leakage more effectively than encapsulation within the inner oil phase. With this regard, Tur-OPE, which encapsulated Tur within the outer particle layer, improved color stability more than inner oil phase encapsulation, thereby making it useful for applications that need to maintain color intensities over a long period [59].

## 4. Conclusions

In this study, we successfully demonstrated the potential of PE stabilized by Ly/CMC NPs to enhance the color intensity and stability of Tur. Ly/CMC NPs were successfully formulated, as affirmed by physical properties and FTIR analysis. Moreover, the oil fraction (φ) of 0.2 was the desirable preparation condition of PE stabilized with Ly/CMC NPs. By comparing encapsulation positions, we illustrated that encapsulation of Tur in OPE significantly exceeded encapsulation in IPE and CE in terms of color vividness. Specifically, Tur was more likely to become more reddish and darker when it was encapsulated within OPE (more like free Tur color value), while it was more likely to become brighter and yellower when it was positioned in IPE and CE. Both methods, positioning Tur in OPE and IPE, successfully maintained the color stability under diverse food processing conditions, such as temperature, pH, and storage. These results demonstrate how this novel encapsulation technique could be employed to overcome the drawbacks of CE systems, especially concerning their color-fading properties and chemical instability. Additionally, by providing a strategy for preserving Tur’s color intensity and retention rate, this approach might imply an important influence on the food industry, where visual appeal is crucial. In summary, the introduction of this position-differentiation strategy in Ly/CMC NPs-stabilized PE suggests a remarkable advancement in chromatic vividness and stabilization of Tur. Moreover, this strategy expands the possible utilization of Tur as a natural colorant in food products that require intense and consistent color stability under various food processing and storage conditions.

## Figures and Tables

**Table 1:** Particle size, PDI, ZP, and EE of Ly/CMC NPs and Tur-Ly/CMC NPs.

	Particle Size (nm)	PDI	ZP (mV)	EE (%)
Ly/CMC NPs	78.2 ± 7.2 ^b^	0.47 ± 0.02 ^a^	−50.0 ± 3.5 ^a^	-
Tur-Ly/CMC NPs	295.4 ± 23.6 ^a^	0.39 ± 0.03 ^b^	−50.1 ± 1.2 ^a^	48.7 ± 2.6

Ly, lysozyme; CMC, sodium carboxymethyl cellulose; Ly/CMC NPs, lysozyme-sodium carboxymethyl cellulose nanoparticles; Tur-Ly/CMC NPs, turmeric oleoresin-loaded lysozyme-sodium carboxymethyl cellulose nanoparticles. Mean ± SD values in the column with different letters are significantly different at *p* < 0.05.

**Table 2:** Effect of heating temperature on retention rate, ΔL*, Δa*, and Δb* values of free Tur and Tur-loaded emulsions.

Temperature (°C)	Sample	Retention Rate (%)	ΔL*	Δa*	Δb*	ΔE
70	Free Tur	65.35 ± 0.49 ^b^	0.17 ± 0.58 ^a^	0.57 ± 0.65 ^a^	2.13 ± 0.64 ^a^	2.34 ± 0.55 ^a^
	Tur-CE	89.11 ± 1.36 ^a^	−0.10 ± 0.06 ^a^	−0.18 ± 0.03 ^b^	0.89 ± 0.12 ^b^	0.92 ± 0.12 ^b^
	Tur-OPE	92.43 ± 1.41 ^a^	0.47 ± 0.08 ^a^	−0.49 ± 0.12 ^b^	0.66 ± 0.17 ^b^	0.97 ± 0.06 ^b^
	Tur-IPE	93.74 ± 1.43 ^a^	0.20 ± 0.29 ^a^	−0.47 ± 0.24 ^b^	0.78 ± 0.05 ^b^	0.98± 0.14 ^b^
90	Free Tur	51.61 ± 2.95 ^b^	1.47 ± 0.85 ^a^	−0.67 ± 0.61 ^a^	3.67 ± 0.32 ^a^	4.08 ± 0.57 ^a^
	Tur-CE	79.32 ± 1.54 ^a^	−0.09 ± 0.07 ^b^	−0.51 ± 0.09 ^a^	1.65 ± 0.19 ^b^	1.73 ± 0.21 ^b^
	Tur-OPE	81.22 ± 1.57 ^a^	1.02 ± 0.27 ^ab^	−0.21 ± 0.04 ^a^	1.43 ± 0.19 ^b^	1.79 ± 0.12 ^b^
	Tur-IPE	83.62 ± 1.62 ^a^	0.19 ± 0.50 ^ab^	−0.57 ± 0.28 ^a^	1.20 ± 0.03 ^b^	1.42 ± 0.20 ^b^

Free Tur, turmeric oleoresin; Tur-CE, turmeric oleoresin-loaded conventional emulsion; Tur-OPE, Pickering emulsion encapsulating turmeric oleoresin in its outer layer; Tur-IPE, Pickering emulsion encapsulating turmeric oleoresin in its inner phase. Mean ± SD values in columns with different letters indicate significant differences between samples at *p* < 0.05.

**Table 3:** Effect of different pH on ΔL*, Δa*, and Δb* values of free Tur and Tur-loaded emulsions.

pH	Sample	ΔL*	Δa*	Δb*	ΔE
3	Free Tur	1.91 ± 0.57 ^a^	−1.28 ± 0.77 ^b^	7.85 ± 1.10 ^a^	8.22 ± 1.20 ^a^
	Tur-CE	−0.52 ± 0.44 ^b^	0.00 ± 0.06 ^a^	0.20 ± 0.20 ^b^	0.59 ± 0.44 ^b^
	Tur-OPE	0.20 ± 0.43 ^b^	0.63 ± 0.25 ^a^	−0.71 ± 0.40 ^b^	1.12 ± 0.19 ^b^
	Tur-IPE	−0.06 ± 0.56 ^b^	0.28 ± 0.06 ^a^	0.13 ± 0.05 ^b^	0.62 ± 0.24 ^b^
5	Free Tur	0.75 ± 0.41 ^a^	−0.62 ± 0.46 ^b^	0.96 ± 0.33 ^a^	1.40 ± 0.66 ^a^
	Tur-CE	−0.35 ± 0.31 ^b^	0.00 ± 0.15 ^ab^	0.31 ± 0.28 ^ab^	0.60 ± 0.16 ^a^
	Tur-OPE	0.15 ± 0.38 ^ab^	0.66 ± 0.27 ^a^	−0.57 ± 0.39 ^b^	1.01 ± 0.34 ^a^
	Tur-IPE	0.00 ± 0.47 ^ab^	0.27 ± 0.04 ^a^	−0.16 ± 0.44 ^b^	0.71 ± 0.18 ^a^
7	Free Tur	1.08 ± 0.42 ^a^	−1.83 ± 0.19 ^c^	0.36 ± 0.43 ^a^	2.24 ± 0.08 ^a^
	Tur-CE	−0.25 ± 0.07 ^b^	−0.16 ± 0.08 ^b^	0.53 ± 0.05 ^a^	0.62 ± 0.05 ^b^
	Tur-OPE	0.24 ± 0.43 ^b^	0.39 ± 0.08 ^a^	−0.27 ± 0.31 ^a^	0.71 ± 0.11 ^b^
	Tur-IPE	−0.06 ± 0.23 ^b^	0.03 ± 0.18 ^ab^	0.19 ± 0.43 ^a^	0.48 ± 0.15 ^b^
9	Free Tur	5.97 ± 1.14 ^a^	−14.49 ± 2.57 ^b^	7.43 ± 1.27 ^a^	17.35 ± 3.07 ^a^
	Tur-CE	0.39 ± 0.46 ^b^	−1.22 ± 0.55 ^a^	1.06 ± 0.53 ^b^	1.73 ± 0.68 ^b^
	Tur-OPE	1.39 ± 0.37 ^b^	−1.32 ± 0.25 ^a^	−0.33 ± 0.32 ^b^	1.97 ± 0.47 ^b^
	Tur-IPE	1.03 ± 0.16 ^b^	−1.29 ± 0.07 ^a^	0.23 ± 0.27 ^b^	1.69 ± 0.15 ^b^

Free Tur, turmeric oleoresin; Tur-CE, turmeric oleoresin-loaded conventional emulsion; Tur-OPE, Pickering emulsion encapsulating turmeric oleoresin in its outer layer; Tur-IPE, Pickering emulsion encapsulating turmeric oleoresin in its inner phase. Mean ± SD values in columns with different letters indicate significant differences between samples at *p* < 0.05.

**Table 4:** ΔL*, Δa*, and Δb* values of free Tur and Tur-loaded emulsions over 4 weeks at 25 °C.

Sample	ΔL*	Δa*	Δb*	ΔE
Free Tur	0.20 ± 0.81 ^a^	1.06 ± 0.81 ^a^	4.22 ± 0.37 ^a^	5.89 ± 0.32 ^a^
Tur-CE	−0.28 ± 0.13 ^a^	−0.39 ± 0.08 ^b^	1.21 ± 0.08 ^b^	1.31 ± 0.13 ^b^
Tur-OPE	−0.67 ± 0.63 ^a^	−0.14 ± 0.59 ^b^	−0.64 ± 0.07 ^b^	1.17 ± 0.16 ^b^
Tur-IPE	0.65 ± 0.12 ^a^	−0.56 ± 0.10 ^b^	−0.33 ± 0.21 ^b^	0.94 ± 0.13 ^b^

Free Tur, turmeric oleoresin; Tur-CE, turmeric oleoresin-loaded conventional emulsion; Tur-OPE, Pickering emulsion encapsulating turmeric oleoresin in its outer layer; Tur-IPE, Pickering emulsion encapsulating turmeric oleoresin in its inner phase. Mean ± SD values in columns with different letters indicate significant differences between samples at *p* < 0.05.

**Table 5:** Kinetic parameters for Tur degradation over 4 weeks of storage in free Tur and Tur-loaded emulsions at 25 °C.

Sample	Time (Week)	Retention Rate (%)	*K* (day^−1^)	t_1/2_ (day)	R^2^
Free Tur	0	100.00 ± 0.00 ^a^	0.0378	18.34	0.9496
	1	62.67 ± 2.68 ^b^			
	2	53.03 ± 5.38 ^c^			
	3	37.41 ± 1.68 ^d^			
	4	34.40 ± 2.32 ^d^			
Tur-CE	0	100.00 ± 0.00 ^a^	0.0069	100.46	0.9615
	1	92.34 ± 3.80 ^b^			
	2	89.15 ± 6.48 ^b^			
	3	87.00 ± 3.15 ^b^			
	4	80.95 ± 10.29 ^b^			
Tur-OPE	0	100.00 ± 0.00 ^a^	0.0042	165.04	0.8839
	1	97.84 ± 4.16 ^a^			
	2	97.17 ± 2.66 ^a^			
	3	93.97 ± 6.95 ^a^			
	4	88.06 ± 6.27 ^a^			
Tur-IPE	0	100.00 ± 0.00 ^a^	0.0051	135.91	0.9645
	1	94.91 ± 1.55 ^ab^			
	2	94.20 ± 2.68 ^bc^			
	3	89.00 ± 2.67 ^cd^			
	4	86.41 ± 2.32 ^d^			

Free Tur, unencapsulated turmeric oleoresin; Tur-CE, turmeric oleoresin-loaded conventional emulsion; Tur-OPE, Pickering emulsion encapsulating turmeric oleoresin in its outer layer; Tur-IPE, Pickering emulsion encapsulating turmeric oleoresin in its inner phase. Mean ± SD values in columns with different letters are significantly different at the same heating temperature with *p* < 0.05.

## Data Availability

The original contributions presented in this study are included in the article. Further inquiries can be directed to the corresponding author.
